# A proposed framework for the systematic review and integrated assessment (SYRINA) of endocrine disrupting chemicals

**DOI:** 10.1186/s12940-016-0156-6

**Published:** 2016-07-14

**Authors:** Laura N. Vandenberg, Marlene Ågerstrand, Anna Beronius, Claire Beausoleil, Åke Bergman, Lisa A. Bero, Carl-Gustaf Bornehag, C. Scott Boyer, Glinda S. Cooper, Ian Cotgreave, David Gee, Philippe Grandjean, Kathryn Z. Guyton, Ulla Hass, Jerrold J. Heindel, Susan Jobling, Karen A. Kidd, Andreas Kortenkamp, Malcolm R. Macleod, Olwenn V. Martin, Ulf Norinder, Martin Scheringer, Kristina A. Thayer, Jorma Toppari, Paul Whaley, Tracey J. Woodruff, Christina Rudén

**Affiliations:** Department of Environmental Health Sciences, University of Massachusetts Amherst School of Public Health & Health Sciences, Amherst, MA USA; Department of Environmental Science and Analytical Chemistry, Stockholm University, Stockholm, Sweden; Institute of Environmental Medicine, Karolinska Institutet, Stockholm, Sweden; ANSES (French Agency for Food, Environmental and Occupational Health Safety), Maisons Alfort, France; Swedish Toxicology Sciences Research Center, Södertälje, Sweden; Charles Perkins Centre, The University of Sydney, Sydney, Australia; Department of health sciences, Karlstad University, Karlstad, Sweden; Icahn School of Medicine at Mount Sinai, New York City, USA; US Environmental Protection Agency, Washington, DC USA; Swedish Toxicology Sciences Research Center (Swetox), Karolinska Institutet, Södertälje, Sweden; Institute of Environment, Health and Societies, Brunel University London, Uxbridge, UK; Department of Environmental Medicine, University of Southern Denmark, Odense, Denmark; International Agency for Research on Cancer, Lyon, France; National Food Institute, Technical University of Denmark, Søborg, Denmark; National Institute of Environmental Health Sciences, Division of Extramural Research and Training, Research Triangle Park, NC USA; Biology Department and Canadian Rivers Institute, University of New Brunswick, Saint John, New Brunswick Canada; Centre for Clinical Brain Sciences, University of Edinburgh, Scotland, UK; Institute for Chemical and Bioengineering, ETH Zürich, Zürich, Switzerland; Department of Health and Human Services, Division of the National Toxicology Program, National Institute of Environmental Health Sciences, National Institutes of Health, Research Triangle Park, NC USA; University of Turku, Turku University Hospital, Turku, Finland; Lancaster Environment Centre, Lancaster University, Lancaster, UK; School of Medicine, Program on Reproductive Health and the Environment, University of California, San Francisco, Oakland, CA USA

**Keywords:** Endocrine disrupting chemicals, Systematic review, Study evaluation, Strength of evidence, Weight of evidence, Adverse effect, Endocrine disrupting activity, Evidence integration, Epidemiology, In vivo

## Abstract

**Background:**

The issue of endocrine disrupting chemicals (EDCs) is receiving wide attention from both the scientific and regulatory communities. Recent analyses of the EDC literature have been criticized for failing to use transparent and objective approaches to draw conclusions about the strength of evidence linking EDC exposures to adverse health or environmental outcomes. Systematic review methodologies are ideal for addressing this issue as they provide transparent and consistent approaches to study selection and evaluation. Objective methods are needed for integrating the multiple streams of evidence (epidemiology, wildlife, laboratory animal, in vitro, and *in silico* data) that are relevant in assessing EDCs.

**Methods:**

We have developed a framework for the systematic review and integrated assessment (SYRINA) of EDC studies. The framework was designed for use with the International Program on Chemical Safety (IPCS) and World Health Organization (WHO) definition of an EDC, which requires appraisal of evidence regarding 1) association between exposure and an adverse effect, 2) association between exposure and endocrine disrupting activity, and 3) a plausible link between the adverse effect and the endocrine disrupting activity.

**Results:**

Building from existing methodologies for evaluating and synthesizing evidence, the SYRINA framework includes seven steps: 1) Formulate the problem; 2) Develop the review protocol; 3) Identify relevant evidence; 4) Evaluate evidence from individual studies; 5) Summarize and evaluate each stream of evidence; 6) Integrate evidence across all streams; 7) Draw conclusions, make recommendations, and evaluate uncertainties. The proposed method is tailored to the IPCS/WHO definition of an EDC but offers flexibility for use in the context of other definitions of EDCs.

**Conclusions:**

When using the SYRINA framework, the overall objective is to provide the evidence base needed to support decision making, including any action to avoid/minimise potential adverse effects of exposures. This framework allows for the evaluation and synthesis of evidence from multiple evidence streams. Finally, a decision regarding regulatory action is not only dependent on the strength of evidence, but also the consequences of action/inaction, e.g. limited or weak evidence may be sufficient to justify action if consequences are serious or irreversible.

**Electronic supplementary material:**

The online version of this article (doi:10.1186/s12940-016-0156-6) contains supplementary material, which is available to authorized users.

## Background

Endocrine disrupting chemicals (EDCs) have received significant attention by scientists across numerous disciplines and risk assessors since the term was first coined in the 1990s [1–5]. Concerns have been raised about associations between EDC exposures and human diseases [6–8], particularly as non-communicable disease rates have risen for many diseases with an endocrine basis [9]. Similarly, studies conducted in laboratory animals indicate that EDC exposures can induce outcomes related to many of these same diseases [3, 10–13].

Since the term ‘endocrine disruptor’ was first used, numerous definitions have been proposed by various groups and agencies. The 2002 report by the International Programme on Chemical Safety and World Health Organization (IPCS/WHO) defined an EDC as “an exogenous substance or mixture that alters function(s) of the endocrine system and consequently causes adverse effects in an intact organism, or its progeny, or (sub)populations” [14]. This definition was used again in the 2012 State of Science on EDCs published by the UN Environment Programme (UNEP) and WHO [15]. Using the major components of the IPCS/WHO and the UNEP/WHO definition of an EDC, identifying a compound as an EDC therefore requires appraisal of:

1. evidence of an (adverse) effect, (remembering that reversible effects might be adverse, there can be a continuum of effects from “initiating events” to “apical effects” induced by the chemical, and that there remains a debate about what should be considered “adverse” outcomes [12, 16, 17])
2. evidence of endocrine disrupting activity (remembering that endocrine disrupting activity extends beyond ‘endocrine active’ compounds and includes disruption to hormone binding, synthesis, secretion, transport and metabolism)
3. evidence of a plausible link between the observed adverse effect and the endocrine disrupting activity

We have focused on the IPCS/WHO definition in this manuscript for a number of reasons. First, the use of this definition by the WHO and other international organizations suggests that it is relevant across the globe. Second, this definition provides a framework for testing, as it is the strictest definition requiring detailed data to address specific points. As discussed later in this manuscript, the selection of other definitions for an EDC may be appropriate in specific contexts, and we provide suggestions for how a decision-making framework would be adapted for these definitions.

Numerous recent reports have summarized the ‘state of the science’ on EDCs [3, 12, 18–22]. These include a 2011 State-of-the-Art Report to the European Commission (SAREC) on EDCs [19] and a report published in February 2013 by UNEP/WHO, entitled “*State of the Science of Endocrine Disrupting Chemicals – 2012*” [15] which was an update of the IPCS/WHO 2002 report, “*Global Assessment of the State of-the-Science of Endocrine Disruptors”* [23]. Using the available evidence, the 2013 UNEP/WHO report [15] drew several key conclusions including: 1) Laboratory studies support the hypothesis that chemical exposures contribute to endocrine disorders in humans and wildlife; 2) Wildlife populations have been affected by endocrine disruption, with negative impacts on growth and reproduction; 3) Internationally agreed and validated test methods for the identification of endocrine disruptors (sometimes called guideline endpoints) capture only a limited range of the known spectrum of endocrine disrupting effects. As a result harmful effects in humans and wildlife may be overlooked; and 4) Disease risk due to EDCs may be significantly underestimated.

## A need for systematic review and integrative assessment criteria

In the 2013 UNEP/WHO report on EDCs [15], the authors discussed the need to develop a structured framework for evaluating evidence of EDC effects, stating “*There is currently no widely agreed system for evaluating the strength of evidence of associations between exposures to chemicals (including EDCs) and adverse health outcomes. The need for developing better approaches for evaluating the strength of evidence, together with improved methods of risk assessment, is widely recognized. Methods for synthesizing the science into evidence-based decisions have been developed and validated in clinical arenas. However, due to differences between environmental and clinical health sciences, the evidence base and decision context of these methods are not applicable to exposures to environmental contaminants, including EDCs.”* Here, we present adaptations to the systematic review approach which do make them applicable in this context.

The term “systematic review” refers to an approach that uses pre-established, consistent and transparent methods to identify and evaluate all available research and information relevant to a research question, topic, or phenomenon [24–28]. The primary goal of systematic reviews is to use transparent, valid and systematically applied criteria to reduce the influence of reviewer bias (in both study selection and study evaluation) and error in the evaluation process [29, 30]. Here, the term ‘transparent’ refers to the open disclosure of the methods to be used, but it is also important to note that ‘transparency’ refers to all aspects of a systematic review, from how the problem statement is formulated, to how the literature is searched, to how data are evaluated and reported. The purpose of systematically evaluating the methodological quality of individual studies is to reliably distinguish along a continuum those that are better and more directly informative from those that are weaker and less directly informative. Systematic reviews follow a set protocol, and though there may be differences in these protocols both within and across fields, they typically include the same key elements (Table 1).

**Table 1:** Key elements of a systematic review protocol

A well-defined study question
A reproducible, transparent literature-search strategy
Pre-determined method(s) used to screen studies based on inclusion/exclusion criteria
A method for evaluation of internal validity of included studies
A summarization of findings from included studies
A method for rating the quality of the evidence across studies
Procedures to synthesize data within individual evidence streams, including decision criteria, using standard terms
Methods to integrate multiple streams of evidence and reach uniform classifications based on objective criteria

Although the use of objective and systematic review methods for identifying, evaluating and integrating evidence is widely accepted in carcinogen hazard evaluation [31], adaptation of methods used in the clinical sciences to other endpoints – including the analysis of EDCs – has only recently started [27, 29, 32]. The need for such analytical tools has been recognized with new methods that have been developed by academic scientists (e.g. the Navigation Guide [29, 30] and SciRAP [33]) as well as the International Agency for Research on Cancer (IARC) and its collaborating experts [34], the European Food Safety Authority (EFSA) and the US National Toxicology Program (e.g. Office of Health Assessment and Translation, OHAT, and the Office of the Report on Carcinogens, ORoC) [28, 35–37]. Detailed descriptions of the development of systematic reviews in the field of environmental health sciences have been provided elsewhere [29, 30, 35, 36, 38, 39].

Systematic review approaches are promoted in regulatory hazard and risk assessment of chemicals in the EU, although guidance for how to conduct such reviews is very limited or even lacking for many groups of chemicals [40]. This lack of guidance potentially hampers consistent and transparent use of systematic review in the risk assessment of chemicals.

One challenge in developing and implementing systematic review methods for EDCs is that information is derived from all levels of investigation – including biochemical and cell-based research, studies of mechanisms and adverse effects in laboratory animals, epidemiological studies and exposure science [41]. (Note that throughout the text, we refer to these broad study designs [epidemiology, laboratory animal, etc.] as different ‘data streams’ or ‘evidence streams’.) Furthermore, the evidence required to conclude that a compound is an EDC, according to the IPCS/WHO definition, requires multiple steps: the chemical must induce an adverse effect *and* there must be a plausible link between that adverse effect and endocrine disrupting activity. Building upon the available systematic review methods in environmental health sciences and adapting them for use specifically for EDCs would expand and improve our capacity to document the strength of the evidence linking EDC exposures and health outcomes. Improved methodologies could also advance research and decision making by shedding light on where the data are sufficiently strong to inform policy decisions and where new data are needed.

In Section III we present a framework for the systematic review of EDC studies. The framework we have developed can be used to identify substances as EDCs according to the IPCS/WHO definition. This process also assesses the strength of evidence associating an EDC with a health or environmental outcome. Each stream of evidence (mechanistic in vitro*,* laboratory animal, ecotoxicology, epidemiology) is evaluated first individually and then collectively, using the principles of toxicology, epidemiology and endocrinology [41]. EDC data are often derived from multiple lines of evidence, and these different types of data need to be evaluated and integrated carefully. Thus, this process is an expansion of methods used in clinical sciences, which typically focus on evidence from human studies, as it incorporates both systematic review and integrated assessment (SYRINA) of different lines of evidence. With regard to EDCs, *the overall objective* of using the SYRINA framework is to provide a sufficient evidence base needed to support decision making, including any action to avoid/minimise potential adverse effects of exposures.

Importantly, the use of the proposed framework allows scientists, clinicians and risk assessors to consider all evidence and knowledge when drawing conclusions rather than identifying so-called ‘key studies’ which is the current principle [42–45]. Further, this framework will allow for transparent application of expert judgement, which has been identified and debated as a critical issue in chemical risk assessments [5, 44, 46]. In systematic review, topic specific expertise is required to develop and implement a high quality protocol. Although expert judgement is unavoidable and plays a critical role in hazard and risk assessment, it inevitably introduces value-based assumptions to the assessment that may influence the conclusions. The use of expert judgement in ways that are not transparent nor consistently applied may increase discrepancies in hazard and risk assessment conclusions, often rendering them irreproducible between experts [47–49]. It is therefore of key importance that any assumptions are transparently described prior to initiating the review to the extent possible. For example, a scientist examining rates of thyroid cancer might focus on the role of thyroid hormone receptor agonists and antagonists while ignoring compounds known to act as anti-androgens, which have no known role in the etiology of this disease; thus, a systematic review might opt to focus only on thyroid disrupting compounds, based on an assumption that compounds with a mechanism of action relevant to androgen signaling do not have relevance to thyroid cancer. This type of a priori assumption should be described transparently so that new information identified in the future that might challenge this assumption can be appropriately considered.

Importantly, our proposed framework is focused on the defintion for EDCs proposed by the IPCS/WHO in that it evaluates both endocrine disrupting actvity and (adverse) effects. Because different definitions and criteria for EDCs are used by authorities in different nations [50], policy and regulatory decisions may only require evidence of endocrine activity *or* (adverse) effects. If other definitions of an EDC are used in decision-making, a revised format of the SYRINA framework proposed here can be used. This is discussed in greater detail later in this manuscript.

Throughout the process, we recommend that reviewers keep in mind and identify the key knowledge and data gaps, uncertainties, and variabilities to establish confidence in the evaluation and to help users of the SYRINA review distinguish between what is known and what is not known. These issues are also discussed in more depth later in the manuscript, but require consideration throughout each step of a systematic review.

## The proposed framework

**Fig. 1:**
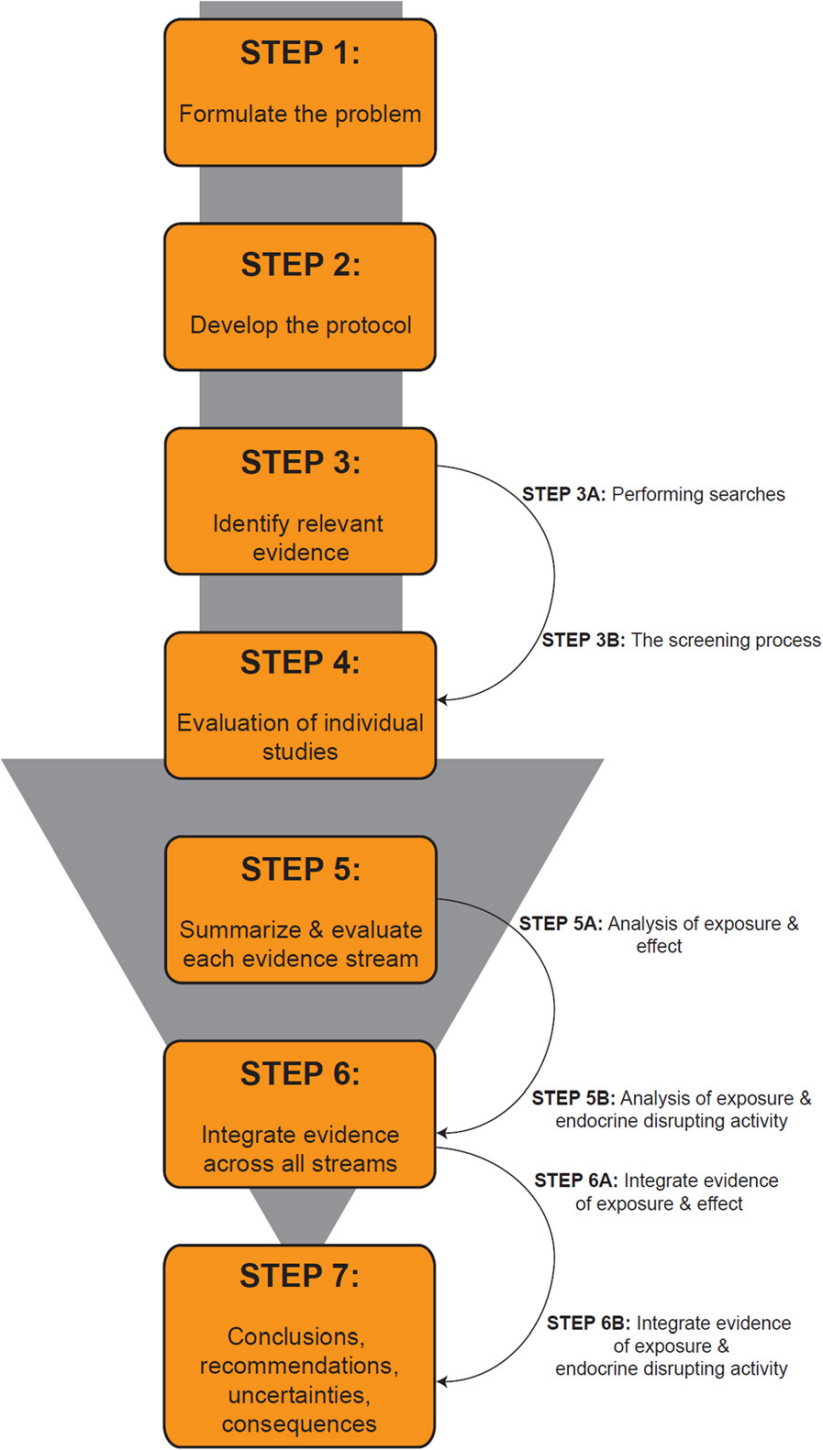
Structure of the proposed framework for the systematic review and integrated assessment of endocrine disruption

We have developed a framework for the SYRINA of environmental chemicals to determine whether they are EDCs and to assess the strength of association between exposure and adverse outcome. This framework, separated into seven steps, is described in detail below and shown in Fig. 1.

### Step 1: Formulate the problem

The first step in the process is to define the overall objective/problem and formulate (an) answerable review question(s). Defining the review question(s) will guide the review process and give the scope of the evaluation. It is important to engage topic-specific experts, with fully disclosed information about potential conflicts of interest, early in this process to provide context and ensure its accuracy, relevance and transparency [44]. Review question(s) should be selected that are feasible, tractable, of high priority, not duplicative, and have a high potential to impact decision-making (considering the time and effort required to conduct a systematic review). Key questions and objectives should also reflect areas of uncertainty. Finally, the objective/problem and review question(s) are formulated as a statement describing Populations of interest, Exposures, Comparators, and Outcomes (PECO) based on the Cochrane PICO statement [27], where the “I” for Intervention is replaced with an “E” for Exposure (Table 2). The quality of exposure assessments is often critical for observational studies. The PECO statement serves as a guide for the entire review process including the literature search strategy, criteria for the inclusion/exclusion of studies, type of data extracted from studies, and strategy for synthesis and reporting of results [38, 51–55].

**Table 2:** Elements of a PECO statement using PFOA and birth weight as an illustrative example

Element	Explanation	Example for perfluorooctanoic acid (PFOA) and birth weight in animals (adapted from [54])	Example for perfluorooctanoic acid (PFOA) and birth weight in humans (adapted from [53])
(P) Population	Condition or disease, characteristics/demographics of the participants. Which setting?, e.g., general population, occupational setting	Laboratory rodents exposed to PFOA in utero, assessed in early postnatal life	Humans that are studied during reproductive/developmental time period (before and/or during pregnancy or development).
(E) Exposures	What are the exposures of interest? What types of chemical(s), what is the timing of exposure that will be considered? Which duration/frequency of exposure or timing of follow-up in relation to exposure?	Exposure to perfluorooctanoic acid (PFOA), CAS# 335-67-1, or its salts prior to mating, or during pregnancy	Exposure to perfluorooctanoic acid (PFOA), CAS# 335-67-1, or its salts during the time before pregnancy and/or during pregnancy for females or directly to foetuses
(C) Comparator	Which exposure groups will be compared to each other (high versus low exposure) (exposure versus control)?	Exposed groups versus vehicle-treated or naïve controls	Humans exposed to lower levels of PFOA than the more highly exposed humans.
(O) Outcome	Which outcomes will be included or covered? Consider adverse effects as well as potential adverse effects.	Body weight during first five days of postnatal development, total litter weight, measures of size such as body length	Effects on fetal growth, birth weight, and/or other measures of size such as length.

Each evidence stream – mechanistic (non-animal, animal or human), laboratory animal, wildlife (epidemiology), and human (epidemiology) – may have its own PECO statement. For example, relevant ages of participants in a human study can be specified under “Population”; specific windows of exposure can be specified under “Exposure”. The important aspect of the PECO statement is that the types of studies to include in the review are made prior to literature identification. These decisions are therefore not determined by the results of the studies, but rather based on what aspects of the study are relevant to answer the overarching question.

The PECO statement will differ depending on the type of review question to be answered as well as the framework being used to assess the compound of interest. For example, a PECO statement might simply ask whether a compound is associated with a specific disease outcome, in which case the mechanism by which the chemical acts need not be evaluated. Alternatively, when the IPCS/WHO definition of an EDC is used as the basis of the PECO statement, separate questions related to the association between the chemical and an (adverse) effect, the association between the chemical and an endocrine mechanism as well as the plausibility of the link between the effect and the endocrine disrupting activity must be addressed [14].

The PECO statement will also be influenced by whether exposures in populations (human, wildlife) are defined and ongoing. For example, when a new compound is produced, it may be possible to evaluate whether some endocrine disrupting properties are identified (and also whether health effects are likely in a population of interest) prior to an actual exposure of that population. In this case, the PECO statement should carefully consider anticipated uses of the chemical, and transparently document the assumptions that were made about potential exposures (routes of exposure, doses, life stages, etc.).

The writing of the PECO statement is perhaps the most important step in the SYRINA framework. How the PECO statement is constructed will influence the depth of the questions that can be answered, and which conclusions will be reached. For example, a PECO statement that focuses on the anti-androgenic actions of a chemical may prevent the assessment of evidence indicating that the chemical interferes with thyroid hormone signalling. For this reason, multiple PECO statements are likely to be needed to fully evaluate the available literature on any select compound.

### Step 2: Develop the protocol

A protocol is a document that lays out the steps of the review in sufficient detail to allow other investigators to repeat the processes described; key elements of the protocol are described in Table 1. Development of a protocol is standard for systematic reviews in clinical medicine [25, 27, 56] and the practice is becoming standard in reviews of preclinical animal data [57–59]. Methods for conducting systematic reviews in environmental health similarly describe the importance of developing a protocol [28, 29, 35, 38]. The protocol serves as the blueprint for the methods that will be used to identify and evaluate the evidence; it provides transparent documentation of the methods to be used, serves as a basis for training reviewers, and reduces bias and errors in the assessment of the literature (since methods are clearly defined, in writing, and developed prior to the start of this assessment rather than basing the review on the findings during the assessment) [27, 38].

The protocol can change during the review process in light of unanticipated issues which only become apparent during data evaluation. Changes to the protocol should be documented at the time that they are made so that they can be disclosed in a transparent manner when the final evaluation is completed. Protocols should also be registered with protocol repositories such as PROSPERO (http://www.crd.york.ac.uk/PROSPERO/) which includes systematic reviews related to environmental health as a demonstration of transparency and open disclosure of the review methods prior to its undertaking [60]. A suggested format for a protocol is given in de Vries et al. [59]. The peer-reviewed open-access journals *Environmental Evidence* (http://environmentalevidencejournal.biomedcentral.com/) and *Evidence Based Preclinical Medicine* (http://onlinelibrary.wiley.com/journal/10.1002/(ISSN)2054-703X) publish systematic review protocols and methodological papers related to the conduct of systematic reviews.

### Step 3: Identify relevant evidence

Within the protocol, a structured framework should provide details for each aspect of the studies to be evaluated; these evaluation criteria provide a basis for deciding which studies are included or excluded.

Exclusion and inclusion criteria are derived from the PECO statement. Results of studies (e.g. whether findings are statistically significant, or “positive” or “negative”) are not appropriate criteria for exclusion or inclusion. Similarly, compliance with standardized test guidelines such as OECD test guidelines, or Good Laboratory Practices (GLP), is not an appropriate criterion for exclusion or inclusion.

**Fig. 2:**
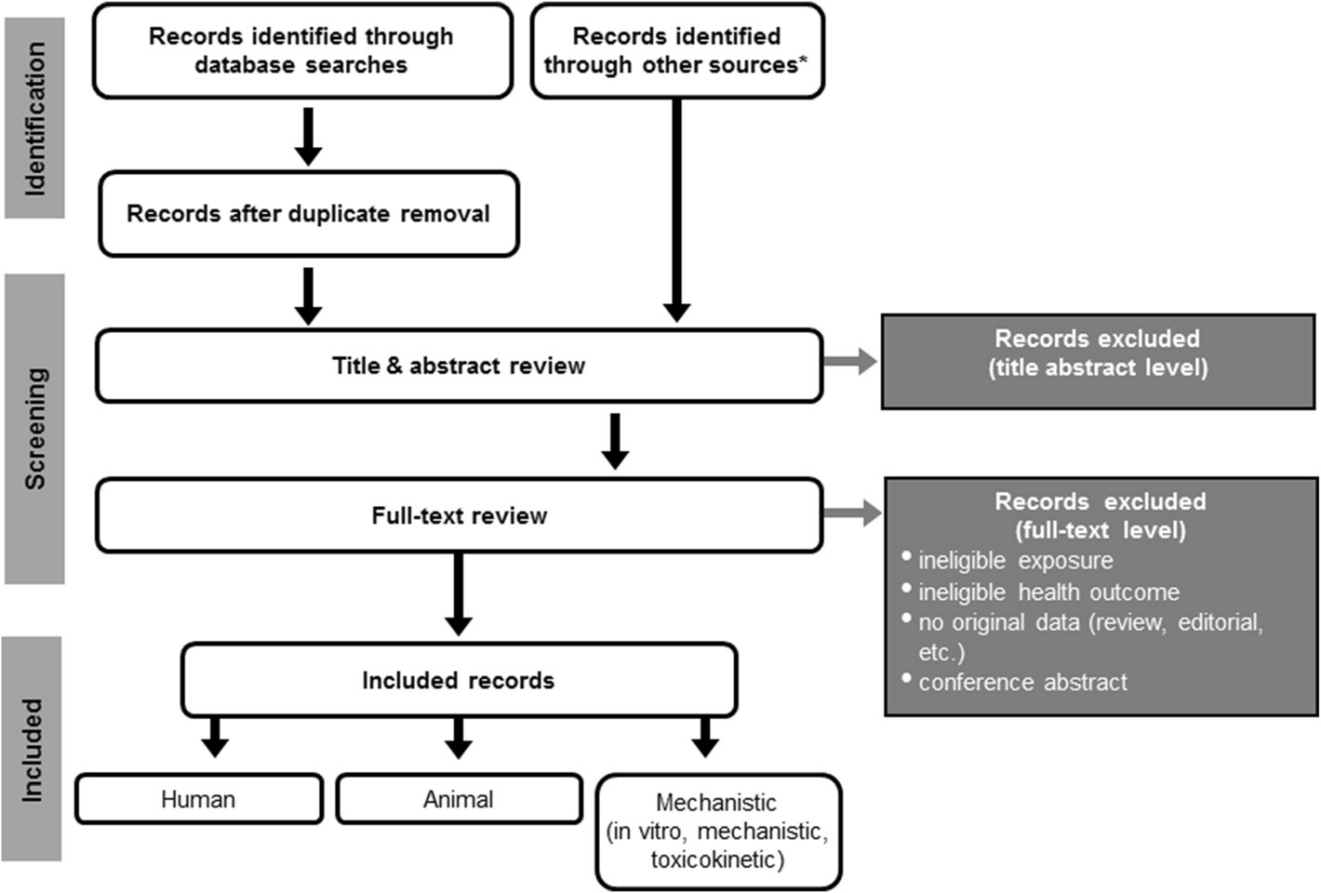
Example study flow diagram

The study flow diagram is a required element of a systematic review that is used to depict the flow of information through the different phases of the evaluation (Fig. 2). It maps out the number of included and excluded records identified, and the primary reasons for exclusions at the full text level [61].

#### Step 3A: Performing searches

The goal of the search strategy is to provide a systematic, objective, reproducible and comprehensive search of the literature relevant to the objective of the review. This is a key feature in clinical systematic reviews and a key step in reducing bias in the overall evaluation [27]. The goal and steps of the search strategy are defined in the protocol. Decisions about the scope of the search are also made, including the databases to be searched and the types of data that will be considered. For example, during the database search stage, decisions should be made about what type of literature should be considered, e.g. peer-reviewed articles, agency reports, consultancy and industry studies, and other proprietary data. Additional file 1: Table S1 outlines data sources that may be relevant when searching for studies related to environmental chemicals. Some have limitations in their search approaches such as character limits and inabilities to support Boolean logic and to export results. Depending on the affiliation of the reviewer, access to these different types of literature may vary. Decisions also have to be made regarding how to address studies written in different languages, studies published only in abstract form, and unpublished results. In general, use of non-English literature is encouraged and has recently become more achievable with online translation tools. Conference abstracts, theses and dissertations may contain relevant data and decisions should be made before starting the review as to whether they will be included. Other sources of information may come from publicly available databases, such as the US National Toxicology Program (NTP)’s Tox21 and US EPA’s ToxCast high-throughput screening platforms. These results may be very relevant to EDCs so they should be considered for inclusion in the protocol.

The literature search should be developed and performed in consultation with a specialist, in particular a trained librarian or a person experienced in systematic review. Others involved in this process include the evaluation team and, as needed, subject matter experts. Typically, the search strategy is developed by: (1) identifying PubMed’s Medical Subject Headings (MeSH) for relevant and appropriate terms, (2) extracting key terminology from relevant reviews and a set of previously identified primary data studies that are known to be relevant to the topic, identified in consultation with the expert team, and (3) reviewing search strategies presented in other reviews. The search strategy may be tailored to make it suitable to other non-PubMed databases such as Web of Science, Scopus, etc. In these circumstances a number of related search strategies might exist, each designed for a specific database.

The search strategy, date of search, and publication dates included in the search should be documented with sufficient detail so that the search can be reproduced, although replication of the exact search results may not be possible as databases change over time. It may be useful to update the literature search at a specified time interval during the evaluation, to capture literature published during the course of the evaluation. This should be defined in the study protocol, perhaps conditionally (e.g. if the review is not completed within 12 months of the search, a further search will be performed). The investigators’ awareness of new research published after the cut-off date for inclusion is not a valid reason for conducting a new search.

Finally, after identifying the studies to be included, the studies’ citations should be evaluated to ascertain if there are any additional relevant records or sources of data that should be included as evidence. Any additional identified studies are then evaluated as below using the same inclusion and exclusion criteria.

#### Step 3B: The screening process

To determine their relevance and eligibility for inclusion or exclusion, articles are independently screened, typically by at least two investigators. There are multiple software platforms that facilitate a more uniform approach to the screening process including DistillerSR®, Rayyan, or Health Assessment Workspace Collaborative (HAWC).^1^ Results from the search are first sorted and any duplicates are removed (e.g. using validated tools for duplicate removal [62]). Next they are reviewed by title and abstract using the developed screening instructions. The goal here is first to screen out any articles that are obviously not relevant to the PECO statement. Next, remaining articles undergo a full-text review using the same screening instructions as for the abstracts. After each step in the process, differences in evaluation between reviewers are identified and resolved via discussion. Machine-learning approaches to prioritizing articles for relevance during screening are also becoming more acceptable for use [63].

At the conclusion of Step 3, the final product is a group of studies that have been deemed applicable to the PECO statement written in Step 1. These studies, selected using transparent and reproducible methods, are then used as the basis for the remainder of the evaluation (Steps 4–7).

### Step 4: Evaluation of individual studies

The purpose of Step 4 is to evaluate the quality of individual studies identified in Step 3. Study quality is a general concept; in the context of evaluating studies for hazard assessment, different definitions of ‘study quality’ have been used across scientific disciplines. Nevertheless, in the context of all disciplines, the purpose of study quality assessment is to evaluate aspects of the study that might influence the interpretation of the results such as the selection, validity, reliability, and/or sensitivity of the methods used.

In systematic review methods developed in the fields of medicine and health care, focus is placed on evaluating a study’s *internal validity. Risk of bias* is one term that has been used for this concept, and is defined as “the extent to which flaws in the design and execution of a collection of studies could bias the estimate of effect for each outcome under study” [64]. Where bias refers to systematic error that reduces validity, risk of bias is the “potential that bias has occurred” [65]. In a recent and comprehensive report, the National Academy of Sciences identified sources of bias in experimental studies (i.e. laboratory animal, in vitro mechanistic) including [65]:

- selection (differences between controls and treated groups at baseline),
- performance (differences in how controls and treated groups are handled throughout the experiment),
- detection (differences in how outcomes in controls and treated groups are assessed), and
- exclusion (differences in how controls and treated groups are removed from the study).

The National Academy of Sciences also identifies sources of bias in observational studies (human epidemiology and wildlife studies) including [65]:

- confounding (differences between the distribution of risk factors between exposure groups, which could occur at baseline or at other points during follow-up),
- measurement (mismeasurement of exposures, outcomes or confounders at any point during the study), and
- reporting (selective reporting of outcomes, analyses, or whole studies)

Studies that have inadequate randomization of subjects or fail to use blinding for participants or evaluators are typically considered to have a high risk of bias.

While the concept “risk of bias” is well known in the fields of medicine and health care, its use is still being introduced into the field of environmental health sciences and chemical risk assessments. In some areas, especially when evaluating experimental data for regulatory risk assessment within the EU, the term *reliability* is commonly used as a descriptor of study quality [66]. In this context, reliability is defined as the inherent quality of the study and is tightly linked to the reliability of the methods used and how the results have been interpreted, as well as to how both methods and results have been reported [66]. It is thus similar to but not as specific as risk of bias.

#### Evaluation of individual studies in each data stream

Numerous tools exist to capture judgements about study quality; these tools were typically designed for the evaluation of either observational studies (human epidemiology and wildlife) or experimental studies (in vivo or in vitro studies). Examples of tools that are applicable for incorporation into the SYRINA are summarized in Additional file 1: Table S2-S5. When selecting one of these tools, it should be applied to the assessment of all studies in each data stream. These tools typically provide tables that can be filled in by evaluators that will summarize each study individually. Below, we summarize approaches for the different types of data streams. We recommend against the use of tools that assign a numerical score for study quality as these can imply a quantitative measure of scientific uncertainty that is misrepresentative.

#### Human epidemiology

Work in evaluating the internal validity of human epidemiology studies in the field of clinical medicine focused on risk of bias in randomized clinical trials [27, 29, 32]. A number of more recent approaches for evaluation of nonrandomized studies of clinical interventions and environmental health studies have also been developed and applied (see Additional file 1: Table S2). One such approach, the “Risk of Bias In Nonrandomized Studies - of Interventions” (ROBINS-I) method, has recently been released [67]; other approaches have been developed by academic groups (i.e., the Navigation Guide) or national or international agencies. Consideration of the predicted direction and magnitude of bias (if possible) is explicitly included in these methods, allowing the appraiser to note whether an observed effect estimate is likely to be an over- or under-estimate of the true effect estimate.

Consideration of the possible roles of bias, confounding and chance in the interpretation of epidemiological studies is an essential component of cancer hazard identification by the IARC and by the US NTP ORoC [31, 37]. These evaluation methods systematically consider a number of aspects of design and analysis to inform the extent to which these factors have been minimized in an individual study. Specific aspects concern the study population, methods used to ascertain disease(s), and to measure exposure; consideration, in design and analysis, of potential confounding variables; other attributes of the analysis that can influence the robustness and interpretation of the results, reporting considerations; and statistical analyses. Emphasis is given to the appropriate use of meta-analyses and pooled analyses to increase precision and to explore potential heterogeneity.

When considering the use of any tool or method for the assessment of EDC epidemiology studies, several important aspects of these methods should be highlighted. First, evaluation of exposure measures is often limited and exposure misclassification is one of the most important issues in epidemiology studies [68]. In addition, a crucial issue with respect to EDC exposures is how well the measure reflects exposure in the etiologically relevant time window for the specific outcome under study. Other aspects of the “sensitivity” component of quality of epidemiological studies (i.e., the ability of the study to detect a true effect of exposure when one exists, similar to the concept of the sensitivity of an assay) may need to be explicitly considered with additional questions and criteria [69]. Potential modification of the bias domains for application to human epidemiology studies are shown in Additional file 1: Table S3.

#### Wildlife studies

Wildlife studies are typically observational in nature, identifying and investigating a disease or disorder and the underlying factors that lead to these conditions. Using study designs similar to those used in human epidemiology, they investigate the presence or absence of associations between exposures and health outcomes on individual wildlife species, and the likely or measured impacts at the population and ecosystem levels. In ecological risk assessments, population-level impacts take precedence over effects observed in individuals. In the current SYRINA framework, observational wildlife studies can be evaluated using the human framework, as the same evaluation of the methods applies. We therefore recommend using the same approach as described above for human epidemiology studies (Additional file 1: Table S3).

Observational wildlife studies may be complemented by experimental studies (e.g. caging, mesocosms, whole ecosystem studies) in which wildlife populations or individuals are intentionally exposed to an EDC or chemical mixture. For these experimental studies, risk of bias and study quality should be assessed using the tools developed for in vivo toxicity studies (see below).

#### In vivo (Mammalian and Non-mammalian) and in vitro toxicity studies

Regulatory agencies and organizations such as the European Chemicals Agency, the US Food and Drug Administration (FDA), and the OECD have promoted the use of the Klimisch method [70] for the evaluation of study quality in toxicity studies. However, there are several challenges with using the Klimisch method, including that 1) no detailed criteria and very little guidance for study evaluation is provided, 2), Klimisch focuses solely on quality of reporting, so that studies conducted according to standardized test guidelines and GLP are by default attributed higher reliability than other research studies and 3) there is no evidence that GLP studies have a lower risk of bias. Reliance on standardized test guidelines is especially problematic in the context of identifying and assessing EDCs since research using novel methods may be more sensitive and relevant for assessing endocrine-related outcomes [12, 42, 45, 71–74]. Comparisons and testing of different evaluation methods, including the Klimisch method, show that there is variability in their development and content, and this affects the outcome of evaluations [45, 74].

The National Academy of Sciences states that “conducting an animal study according to [GLP], or by complying with human-subjects guidelines for a clinical study” ensures that the research was conducted using high standards [65]. However, the National Academy of Sciences also notes that this consideration does not represent the totality of what should be included in the evaluation of individual studies. We note that GLP is a method for ensuring adherence to protocols and study reporting, and does not ensure that a study had a high-quality design. There are clear examples of how a study could follow GLP but fail to consider these other important aspects of study design (e.g. contamination of a negative control group, inappropriate laboratory technique, improper reporting of animal ages, failure of a response in a positive control group, etc. [43, 75–77]); in these types of circumstances, these studies should not be deemed high quality.

Several new evaluation methods have been developed that better target internal validity of in vivo or in vitro toxicity studies, and provide more structured support for determining a study’s adequacy for hazard and risk assessments. Additional file 1: Table S4 presents an overview of recently developed evaluation methods for in vivo toxicity studies (including non-mammalian aquatic species) that could be used for EDCs. These methods offer detailed criteria and guidance to help risk assessors and others conducting SYRINAs to make use of all available studies. In addition, the methods promote increased transparency and structure of the evaluation process. Relatively few methods have been developed for the evaluation of in vitro toxicity studies (Additional file 1: Table S5), and only one tool has undergone pilot testing.

#### In silico data

For the purpose of this review, *in silico* methods are limited to those that predict the potential for a particular chemical structure to cause “endocrine disruption”. These often include computational methods that derive structure-activity relationships (SAR) and thus predict potential EDC activity for a given chemical structure. SAR methods can also provide an understanding of the variations in chemical structures that contribute to variations in EDC activity. Table 3 summarizes our recommendations regarding requirements to evaluate *in silico* data. The REACH legislation provides guidance for use of quantitative SAR (QSAR) models [78], dictating conditions for the underlying mechanism, the modelling method and the assessment of reliability. The OECD also provides guidance on how to evaluate *in silico* data [79].

**Table 3:** Evaluation methods for *in silico* models

Modeling type	Description of the method
For all modeling work:	▪ Standardization and curation of the investigated dataset to ensure consistency. This should include a clearly-stated method (including inclusion and exclusion criteria) for curation of the data and a review of the rules applied to chemical structures in order to ensure standardization
QSAR models:	▪ Use of sufficiently diverse training set covering the EDC compound domain of interest
	▪ Use of sufficiently diverse external test set covering the EDC compound domain of interest should be used
	▪ Assembly of internal and external validation, i.e. several internal and external validation sets, and models created in a double loop fashion, followed by consensus predictions
	▪ Sufficient statistical quality achieved
	▪ Consistent applicability domain established, e.g. using a conformal prediction framework
For ligand based pharmacophore models:	▪ Use of sufficiently diverse training set covering the EDC compound mechanism/domain of interest
	▪ All training set compounds should, approximately, fit the derived model equally well unless there are demonstrable differences in the binding affinity
	▪ Use of sufficiently diverse external test set that covers the EDC compound domain of interest to demonstrate generalizability
Protein structure based models:	▪ Several protein structures should be used to account for flexibility of the protein covering relevant conformations
	▪ Use of sufficiently diverse training set covering the EDC compound domain of interest
	▪ Consensus docking and scoring to ensure robustness and stability of results
	▪ Use of sufficiently diverse external test set covering the EDC compound domain of interest

### Step 5: Summarize and evaluate strength of each stream of evidence

The development of the SYRINA framework considered the viewpoints proposed by Austin Bradford Hill [80], which were proposed within the context of evaluation of epidemiology studies. Importantly, many systematic review methods have adopted the approach of GRADE, which provides guidance on how to utilize a structured framework for assessing overall quality of the evidence. As discussed below, the factors considered by GRADE overlap with many of the Bradford Hill considerations but provide guidance on how to operationalize and are expanded to include publication bias [81]. The Cochrane Collaboration has adopted the principles of the GRADE system for evaluating the quality of evidence for outcomes reported in systematic reviews.

The purpose of this step is to summarize relevant data from the individual studies evaluated in Step 4, and to synthesize the evidence within each stream (e.g., human epidemiology, wildlife, laboratory animal, in vitro, *in silico*). If using the IPCS/WHO definition of an EDC [14], two types of evaluations should occur during this step: each stream of evidence is evaluated for the question of “strength of evidence for [pre-determined] effect” and then again for “strength of evidence for endocrine disrupting activity”. In this case, at the end of this step, the evidence within each stream will be characterized by a pre-defined descriptor (see Table 4 for examples) describing the confidence in the association or strength of evidence pertaining to each of two relationships:

**Table 4:** Example descriptors that can be used to characterize confidence in the strength of the evidence between two factors (like exposure and adverse outcomes) within a data stream

Descriptor	Explanation
High	New research is unlikely to change the conclusions drawn from the currently available studies; conclusions are based on a set of studies in which chance, bias, confounding and other alternative explanations can reasonably be ruled out.
Medium	New research could affect the interpretation of the findings. Conclusions are based on a set of studies in which chance, bias, confounding or other alternative explanations cannot reasonably be ruled out as explanations.
Low	The available studies do not allow an inference regarding toxicity because of limitations such as inadequate sensitivity or relevance of the study designs.
Absent	No studies available.

- Association between chemical exposure and (adverse) effect (Step 5a)
- Association between the chemical and endocrine disrupting activity (Step 5b)

The plausibility of the link between these two factors, as required by the IPCS/WHO definition of an EDC, is assessed in Step 7.

#### Step 5A: Analysis of the strength of association between exposure and (adverse) effect within evidence streams

In this step, the evaluation of studies from Step 4 is combined with additional considerations to draw conclusions about the strength of evidence within each stream. Approaches have been developed and applied by the OHAT/NTP and the Navigation Guide and integrate best practices used in evaluating overall evidence from IARC and US EPA [82]. These have been recently reviewed by the National Academy of Sciences [65].

In methods based on GRADE (e.g. the Navigation Guide and OHAT approaches [25, 83]) the strength of evidence from each stream is assessed in two stages. An initial confidence rating is modified by aspects of quality (e.g., risk of bias considerations) and other factors that can lower confidence (inconsistency, indirectness, imprecision, or publication bias) or raise confidence (magnitude of the effect, dose–response gradient, direction and impact of residual plausible confounding, and consistency across evidence streams). One way to assess consistency is to generate visual displays of the results across studies. Several types of displays can be useful, including exposure- (or dose-) response graphs depicting effect size in relation to exposure level in one or more studies, and forest plots that typically depict effect size for each study in a set of studies [84]. Although forest plots do not depict exposure level along an “x axis”, studies can be grouped by various factors, such as exposure level, to examine patterns. The process of sorting and grouping, in particular, stratification by quality assessment (overall, and for specific risk of bias domains) or by exposure level, is the foundation for examining the question of whether reasonable explanations can be made for the patterns seen across studies. Analysis of these factors results in confidence or strength of evidence rating categories for each stream: “high”, “medium”, “low” and “absent” (i.e. no studies available) (Table 4).

We recommend that the initial rating for experimental laboratory animal studies should be set as “high” based on the approach in clinical medicine, where randomized clinical trials (i.e. controlled and randomized exposure studies with the inclusion of a relevant control group) are rated “high”. For epidemiology studies, the choice of the initial confidence rating for the GRADE-based methods is an issue that is undergoing research and review. “Medium”, has been recommended for an initial rating of observational (epidemiology) studies [30, 85]; another option is to start all studies as high (i.e., the top rating level); this rating could subsequently be modified by the risk of bias evaluation. The National Toxicology Program ORoC bases the initial confidence rating on the evaluation of the studies (Step 4) rather than on an a priori designation for all studies of a given design. The evaluation process results in an overall rating for the confidence (or risk of bias) for each study. Thus, for example, results from a set of high confidence would be given an initial confidence rating of high. The confidence level is then modified based on considerations that overlap with those used by the National Toxicology Program OHAT and Navigation Guide (Additional file 1: Figure S1). Results from low confidence (high risk of bias) studies can supplement, but would not negate or override the conclusions drawn from higher confidence studies.

Following this initial rating, evidence streams are up- or down-graded according to specified features of the body of evidence. The following factors should be considered to downgrade confidence in a stream of evidence:

- limitations in methodological quality of the research in the stream (including risk of bias across studies)
- important and unexplained inconsistency in study results within the stream
- imprecise or sparse data

The following factors can be considered to upgrade confidence in a stream of evidence:

- large magnitude of effect
- consistency across different study designs or species
- dose response gradients observed in similar studies
- confounding is minimized

These adjustment factors are not scored numerically [27] but should be considered transparently as part of the process and are evaluated as to whether overall rating should be upgraded or downgraded.

As part of the analysis of consistency and to aid in the interpretation of the quality of the evidence, if possible, a meta-analysis of some or all of the studies within a stream of evidence should be conducted to calculate a summary effect estimate. This summary estimate can provide a more precise estimate of the magnitude of effect than an estimate from an individual study. For the purpose of hazard identification, however, a single effect estimate may not be needed, as the focus is on examining patterns and variability (consistency) across studies. If a meta-analysis is conducted, the validity of the assumption that the studies are examining a common effect estimate must be carefully considered; this consideration requires more than a statistical test of heterogeneity [85, 86]. Study quality, exposure level, exposure route, species, and numerous other considerations may contribute to the observed results and to heterogeneity among studies. If a meta-analysis is conducted, the synthesis must also include a discussion of the results from studies that did not contribute to the combined analysis, for example because their results could not be converted into the form used in the meta-analysis.

After conducting this process, a confidence descriptor (e.g., strong, moderate, weak, absent) is generated for the *strength of evidence* for each evidence stream. Confidence descriptors can also be used to summarize studies that conclude that no effect was observed. The confidence descriptor produced in this step designates conclusions related to the association between exposure and adverse effects: e.g. *“We have high/medium/low confidence that exposure to compound X is associated with adverse outcome Y in humans (or causes outcome Y in animals).”* Confidence descriptors can also be used to summarize studies that indicate that no effect was observed. Confidence descriptors should not be used to characterize the strength of the effect associated with exposure to the compound (e.g. it is not appropriate to use this evaluative tool to say, *Chemical X has a strong/moderate/weak ability to produce adverse outcome Y.*).

#### Step 5B: Analysis of the strength of association between the chemical and endocrine disrupting activity within streams

A similar process to that described in Step 5A can be used for the synthesis of studies aimed at addressing endocrine mechanisms; a set of considerations are applied to conclusions drawn from a group of studies. Again, we propose the same four-level rating system to summarize the strength of evidence in each stream: “high”, “medium”, “low” and “absent” (no studies available) (Table 4).

After conducting this process, a confidence descriptor (e.g., high, medium, low, absent) is again generated for the strength of evidence for each evidence stream, but this descriptor is related to the association between the chemical and endocrine disrupting activity: e.g. *“We have low/medium/high confidence that compound X is associated with Y endocrine disrupting activity in experimental animal studies.”*

### Step 6: Integrate evidence across all streams

The purpose of this step is to integrate the streams of evidence that were assessed in Steps 5A and 5B to come to a conclusion about the overall strength of the evidence for endocrine disruption. First, the individual streams of evidence need to be integrated to assess the strength of the evidence relating to the association between exposure and an adverse effect (Step 6A). Second, the strength of the evidence for each of the conclusions from Steps 5A and 5B are integrated to reach a conclusion about endocrine disrupting activity (Step 6B).

#### Step 6A: Integration of evidence: outcome/adverse effect

**Fig. 3:**
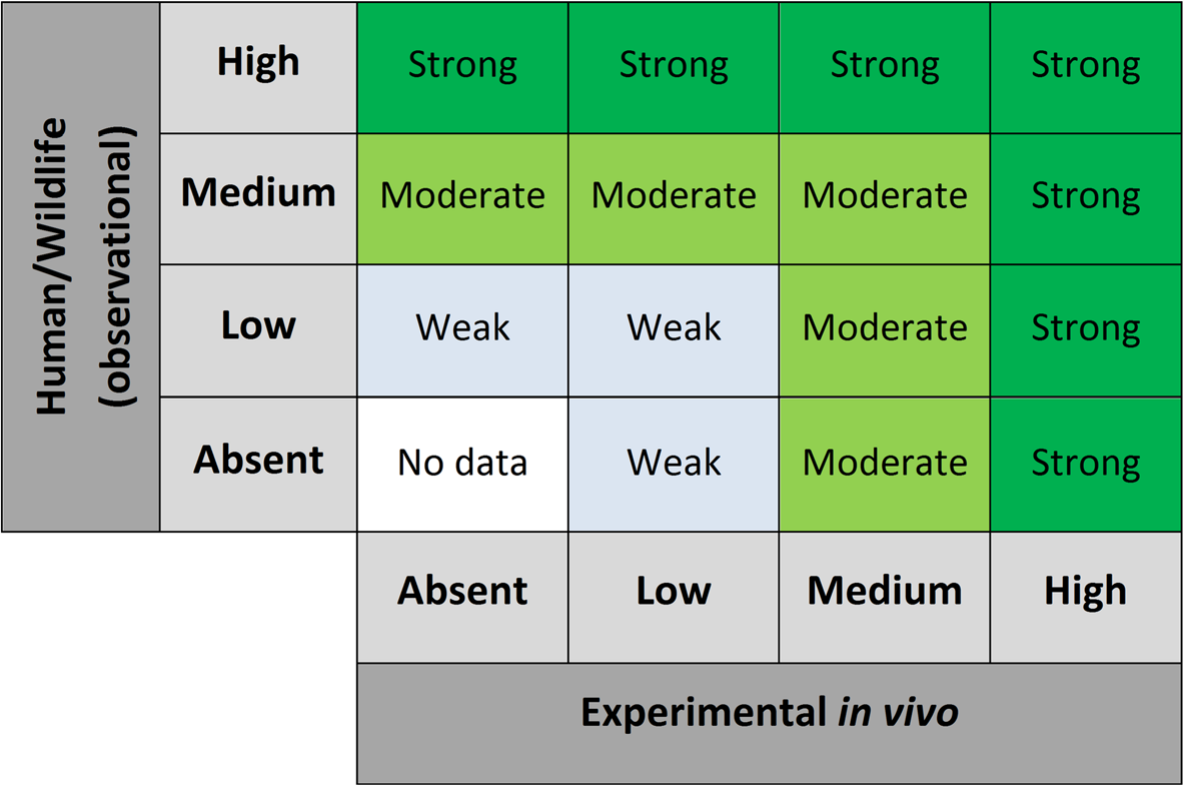
Determining the strength of the evidence for the association between exposures and (adverse) effect. Evidence is characterized as “strong”, “moderate”, “weak,” or “no data”. If in vitro or *in silico* data is considered strong, upgrade “weak” to “moderate”, or “moderate” to “strong”

The aim of this step is to assess the overall strength of evidence that exposure to a potential endocrine disruptor is associated with a health outcome. This is a function of the combined certainty of each stream of evidence. The first step in the process is to use a matrix (Fig. 3) to assign an initial value to the strength of the evidence acquired from observational evidence streams (i.e. human or wildlife studies) and experimental in vivo evidence streams. Based on the combined strength of the observational and experimental streams, the strength of the evidence for the association between exposure to the substance and a health outcome is characterized as “strong”, “moderate”, “weak,” or “no data”. Here, the overall strength of the evidence achieved from integrating multiple streams of evidence will be at least as high as the highest strength of evidence obtained for any single stream. This value can be adjusted up one step, i.e. from “weak” to “moderate” or from “moderate” to “strong”, if there is high confidence in the evidence from *in silico* and in vitro studies. Explanations of the terms “strong”, “moderate” and “weak” should be developed as part of the systematic review framework.

Most importantly, the result of this assessment can potentially yield a health hazard classification independent of any endocrine disrupting effects of the compound under review, i.e. this step can allow for a conclusion that *“We have strong evidence that exposure to compound X causes adverse outcome Y”* even if no information about endocrine disrupting properties of compound X is available. Outside of the framework of the IPCS definition of an EDC, it may not be necessary to identify the mechanism by which a chemical acts prior to regulating its use. Thus, a systematic review that solely identifies the strength of evidence linking a compound to a health outcome may be sufficient to implement a public health response.

#### Step 6B: Integration of evidence: endocrine disrupting activity

**Fig. 4:**
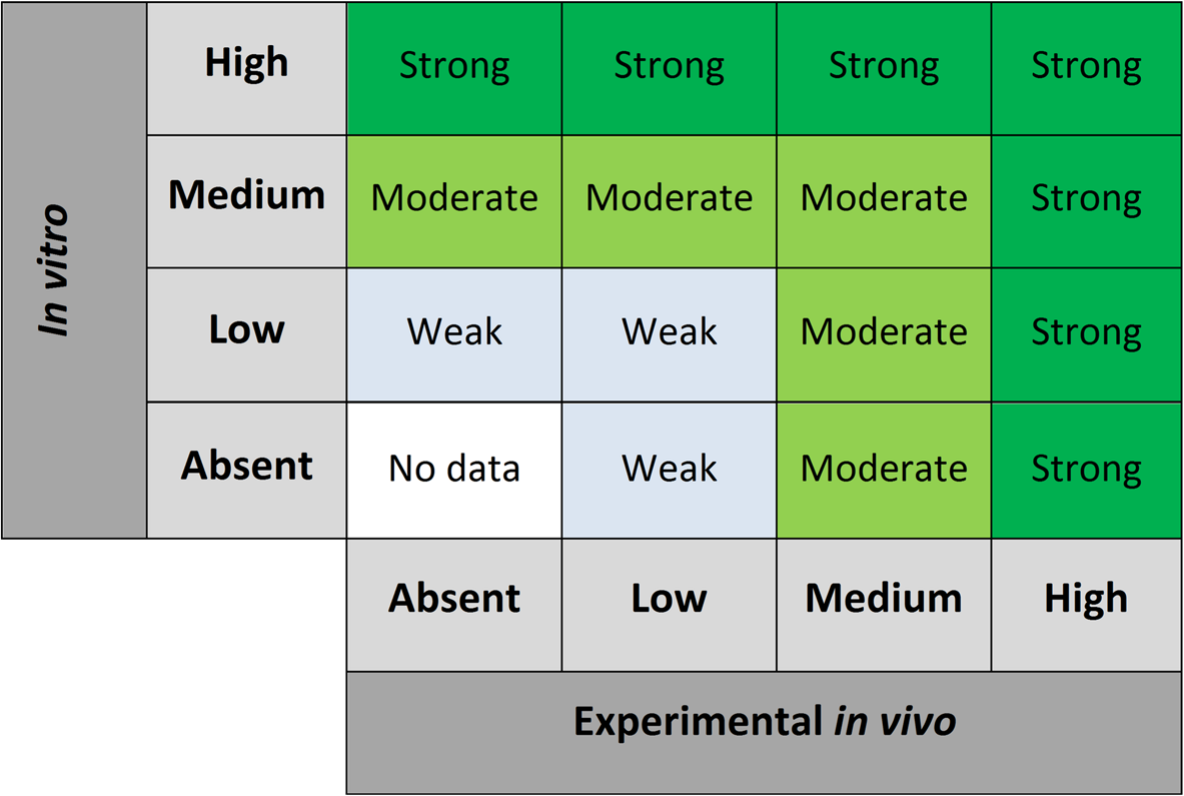
Determining the strength of the evidence for the endocrine disrupting activity of a chemical. Evidence is characterized as “strong”, “moderate”, “weak,” or “no data”. If observational or *in silico* data is considered strong, upgrade “weak” to “moderate”, or “moderate” to “strong”

The main aim of integration at this stage is to qualitatively assess evidence of endocrine disrupting activity. In Step 5, individual streams of evidence were evaluated and assessed with respect to this feature. Again, the overall strength of the evidence achieved from integrating multiple streams of evidence will be at least as high as the highest strength of evidence obtained for any single stream (Fig. 4). The same classifiers are used to describe the strength of data in this step as in Step 6A (Table 5).

**Table 5:** Example descriptors that can be used to characterize confidence in the strength of the evidence after integration across data streams

Descriptor	Explanation
Strong	Future research might make estimates of effect size more precise but are unlikely to show these findings to be a false positive.
Moderate	Although the evidence might be suggestive of an effect, overall it cannot be judged with any confidence whether this effect is real or not; future research may show this to be a false positive.
Weak	There is insufficient evidence for inferring that exposure to the compound is associated with the (adverse) effect. Importantly, we note that this is not equivalent to inferring that the compound is not associated with the (adverse) effect.
No data	No studies available.

### Step 7: Conclusions, recommendations, uncertainties and consequences

#### Drawing conclusions: the IPCS/WHO definition of an EDC

**Fig. 5:**
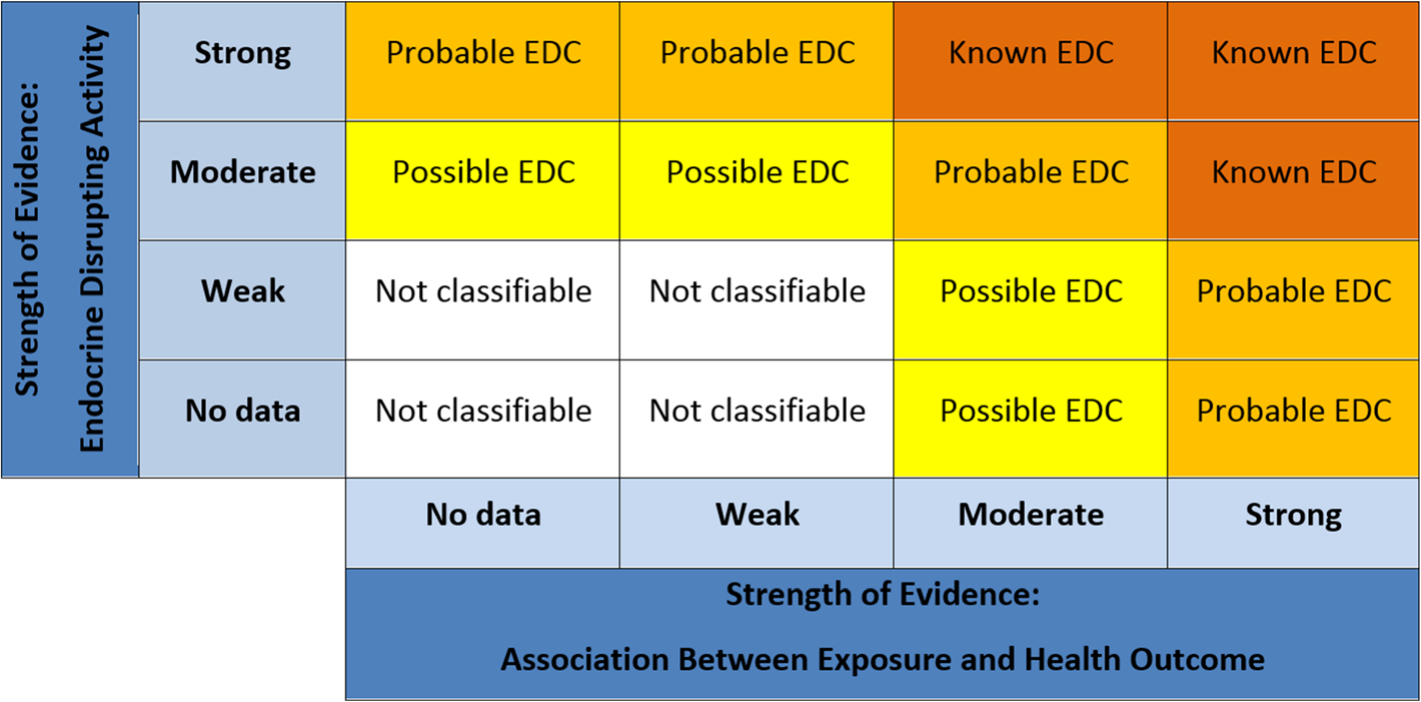
Matrix for drawing conclusions about endocrine disruption. Note: “not classifiable” does not mean that it is not an EDC, simply that not enough data is available to draw a conclusion

The IPCS/WHO definition of an EDC requires an integration of: 1) the health outcome; 2) the endocrine activity; and 3) the plausibility of the link between the outcome and endocrine activity. To complete the SYRINA process, a final integration step can be accomplished using a matrix (Fig. 5). Confidence levels generated in Steps 6A and 6B for evidence of the outcome and evidence of the endocrine disrupting activity are combined to reach preliminary conclusions for the strength of the evidence that a compound is an EDC.

In this final step, consideration is given to the plausibility of the link between endocrine disrupting activity and outcome. The strength of the link can be used in relatively unusual cases to up- or down-grade the preliminary conclusions. Situations where the preliminary conclusions can be downgraded include those where the outcome is multifactorial (e.g., breast cancer) and the evidence for the endocrine disrupting activity (e.g., estrogenicity) is only ‘probable’ or ‘possible’. Similarly, it may be appropriate to upgrade the preliminary conclusion when the evidence of an outcome (e.g., decreased male anogenital distance) has an acknowledged strong link to endocrine activity, but data are limited to support conclusions on endocrine activity for that particular chemical.

The objective of a SYRINA is to provide a scientifically supported evidence base for others to act in order to avoid or minimise potential adverse effects, rather than waiting to observe adverse effects in humans or the environment before any actions are taken. The conclusions of a SYRINA will therefore include statements about *potential* hazards. Based on the results obtained during Step 6, these conclusions will usually take the form of statements such as *“Chemical A is a known/probable/possible endocrine disrupting chemical based on strong/moderate/weak evidence from human and non-human studies.”*

As noted in Step 1, the conclusions that will be reached about the likelihood that a chemical is an EDC will depend on how the PECO statement is framed. For this reason, we caution against drawing overly broad conclusions about the lack of endocrine disrupting properties of a chemical based on the results of a narrow analysis; using the SYRINA framework, one could conclude that a chemical is not classifiable as an EDC based on one PECO statement but could conclude that the same chemical is a known EDC based on another PECO statement that evaluates different literature. For example, a review that is focused on the anti-androgenic effects of a suspected EDC would likely not assess the same literature as a review focused on the thyroid hormone disrupting properties of the same compound.

#### Drawing conclusions outside of the IPCS/WHO framework

The SYRINA framework described here was designed specifically to address evidence of endocrine disruption as it is defined by the IPCS/WHO [14]. Yet, there are numerous other definitions of an EDC used by other decision makers [50]. In contrast with our proposed framework, some of these other definitions do not require that health outcomes, endocrine disrupting activity and the plausibility of the link between the two are all considered separately before a conclusion can be made [12, 16, 50]. Thus, if using these alternative definitions, a modified version of our SYRINA could be successfully employed. For example, evaluating exposures and health outcomes would be consistent with many regulatory and policy needs and is consistent with other systematic review approaches. The overall structure of the systematic review would remain the same (Fig. 1), but Steps 5 and 6 would be simplified.

It is important to note that, regardless of which definition is used, the use of the SYRINA framework requires participation from individuals with a range of expertise. Not only are experts in aspects of study design for each evidence stream needed, scientific experts that understand the biological processes implicated in the PECO statement are needed (e.g. PECO statements related to the risk of breast cancer require experts in mammary gland biology, cancer, and other scientific fields); generalized toxicology or endocrinology knowledge is not likely to be sufficient. Without such subject matter experts, the conclusions that are drawn may be incomplete or inaccurate.

### Making recommendations

The *evaluation of evidence* about the potentially adverse effects of an EDC is a different activity from deciding *how to act* on the basis of that evidence. Who evaluates the evidence and who then recommends action, based on the available evidence, depends on the issues and practices involved in different domains of activity and regulatory contexts. In many regulatory agencies tasked with addressing chemical safety, the evaluation of evidence is performed by “risk assessors” whereas the recommendations for action are made by “risk managers”. Recommendations are typically based on both the evidence of risk (or hazards), and on the availability, feasibility, and cost of the options for action.

As Bradford Hill and others have addressed, there is no direct link between the strength of evidence about a threat (e.g. a chemical’s hazard) and the strength of recommendations about how to minimise or avoid it. For example, information may be available only from epidemiology studies with designs in which bias and confounding may be impossible to resolve, yet an intervention may be strongly recommended given the serious consequences of inaction*.*

Some groups may prefer to use a modified version of the SYRINA framework where direct evidence for *mechanism of action* is not required (e.g. in a modified SYRINA, decisions could be made based solely on the evidence linking a chemical to a harmful outcome, rather than a requirement that the compound be demonstrated to act via and endocrine mechanism). Mechanistic data can take decades to collect and to be agreed upon by experts in the field [44, 87]. Decision makers are faced with choices as to when and where to act on the endocrine disrupting related causal pathway from effects to adverse effects, given the need to act before harm to human health or environments arises, especially if it is irreversible and or trans-generational.

### Handling uncertainties

In each step of the SYRINA, as in the assessment of chemical hazards and risk in general, there are sources of scientific uncertainties and the possibility for error that could contribute to false-positive or false-negative conclusions. There are, for example, uncertainties associated with assumptions made while formulating the problem or review question, as well as how the PECO statement is framed. Uncertainties also arise when extrapolating from data that is only indirectly relevant to the target population, e.g. toxicity data in other species, or exposure scenarios that differ from actual exposure. Incomplete or lack of data also contribute to uncertainties in the final conclusions.

A specific source of uncertainty is the reliance on “statistics” in individual studies to make conclusions about the strength of evidence. “Statistical significance” is generally achieved when the probability *p* that the study results deviate from expectation under the null hypothesis is less than a limit of 5 %. However, this limit may rule out many potentially causal associations, e.g. because the study was too small to reach statistical significance [88]. While aiming to avoid bias toward the null (e.g. false negatives), the risk of false positives should also be considered. There is an ongoing debate over the prevalence of false positives in some fields of biomedical research [89, 90]. Publication bias is always a concern, but endocrine disruption would seem less vulnerable, as the ratio of true to no relationship among the relationships probed in published studies is likely much higher than in most other fields [87]. For example, only a few percent of industrial chemicals in use in the late 1970s were considered hazardous, while that was true for about 70 % of new chemicals tested [91]. The “untested chemicals assumption” therefore causes a very large proportion of false *negative* conclusions [92]. In contrast, when scrutinising alleged false *positive* findings in environmental health and toxicology, very few such cases have been found [93, 94]. Thus, the impact of publication bias would therefore be negligible in comparison with the false negatives due to the huge number of chemicals, for which virtually no information exists on endocrine disrupting properties.

Sources of uncertainty related to any of the steps in the systematic review should be disclosed and characterized as far as possible in order to inform risk managers. Guidance for evaluating and expressing uncertainty in hazard and risk assessment is available from the International Programme on Chemical Safety (IPCS) [95]. Guidance for handling uncertainties in scientific assessments is also under development at EFSA.

### Evaluating consequences

There is often an intermediate step between evidence evaluation/integration and taking action and this is the evaluation of the consequences of being wrong about either the evaluation or the threat. For example, at this stage it is important to consider the consequences of being wrong about a chemical being hazardous, or the consequences of a failure to act following evidence that an EDC is hazardous. As the consequences of being wrong in both cases can be serious, and sometimes irreversible, they may need to be evaluated and reported by evidence evaluators whose expertise contributes to such consequence analyses.

The plausibility and likelihood of being wrong with an evaluation of evidence is related to the *confidence* that evaluators have placed in their conclusions. We propose that this confidence can be increased by using transparent and reproducible methods that acknowledge uncertainties during the process of completing a SYRINA.

## Future research needs

The SYRINA framework described in this manuscript provides methods to critically evaluate scientific literature. Like the methods on which it is based (OHAT and Navigation Guide), there is a need to explore and evaluate the best use of these tools in hazard identification and regulatory contexts and from these experiences, and improve these methods for greater efficiency and transparency. Several areas have been identified in this review as short-term needs including the development of methods for evaluating in vitro and *in silico* data. We note the need for a case study which would also shed light on important procedural issues, including the number and types of experts needed to evaluate evidence from different scientific fields. Such a case study would also be important to identify the time required to complete Steps 1–6 of SYRINA based on examples with many relevant studies versus other examples with minimal available data. We anticipate that the completion of Steps 1–6 could take more than a year to conduct if a relatively large dataset is identified; importantly, this could lead to further delays in the already lengthy processes for regulatory decision-making and other public health oriented actions. We also note that some risk assessment groups will need to make concrete decisions about how PECO statements are written and which risk of bias tools are used in evaluation of studies within evidence streams, as this level of consistency is needed to produce harmonized evaluations.

In fall 2015, the US National Academy of Sciences convened a panel specifically to evaluate the data in support of “low dose effects” for environmental chemicals including EDCs. (For narrative reviews of the ‘low dose’ literature, see [96, 97].) The National Academy panel specifically aims to use systematic reviews to evaluate the published literature, and therefore might benefit from use of the SYRINA framework. Work from this panel will continue through 2016, and may include one or more case studies, providing additional evidence about the feasibility of systematic reviews with broad goals [41].

The IPCS/WHO definition of an EDC raises another important future research need: the delineation of effects as adverse (or not) has been a source of significant debate [16, 17]. In the absence of a definition of ‘adverse effects’ by many risk agencies, decisions on adversity are left to the discretion of individuals. This application of expert judgement is rarely transparent, which is problematic in the context of a systematic review.

## Conclusions

A number of recent analyses of the EDC literature were criticized for failing to use systematic review methods to draw their conclusions. Importantly, systematic review processes specific to EDCs were not yet available for use in these reports. Here, we have developed a seven-step framework for the systematic review and integrated assessment of EDC studies; this framework has a direct application to the IPCS/WHO definition for EDCs. It is also amenable for use in the context of other definitions of an EDC or, with some minor modifications, it could be used to conduct systematic reviews of environmental chemicals that are not EDCs. The SYRINA framework can be used in hazard assessment, which encompasses the first step of the risk assessment process.

The implementation of the SYRINA framework will enhance the assessment of the EDC literature, allowing for transparent and reproducible analyses to be conducted. Case studies using this framework are needed to demonstrate its use and identify any flaws. Importantly, frameworks for systematic reviews should be robust enough to adapt to future needs, and the SYRINA framework was designed with this objective in mind.

## Abbreviations

DRAGON, Dose Response Analytical Generator and Organizational Network; EDC, endocrine disrupting chemicals; FDA, Food and Drug Administration; GLP, Good Laboratory Practices; HAWC, Health Assessment Workspace Collaborative; IARC, International Agency for Research on Cancer; IPCS, International Program on Chemical Safety; MeSH, Medical Subject Headings; NTP, National Toxicology Program; OECD, Organization for Economic Cooperation and Development; OHAT, Office of Health Assessment and Translation; ORoC, Office of the Report on Carcinogens; PECO, Populations, Exposures, Comparators, Outcomes; PICO, Populations, Interventions, Comparators, Outcomes; QSAR, quantitative structure-activity relationship; ROBINS-I, Risk of Bias In Nonrandomized Studies - of Interventions; SAR, structure-activity relationships; SAREC, State-of-the-Art Report to the European Commission; SciRAP, Science in Risk Assessment and Policy; SYRINA, systematic review and integrated assessment; UNEP, United Nations Environment Programme; US EPA, US Environmental Protection Agency; WHO, World Health Organization

## Additional file

Additional file 1: A proposed framework for the systematic review and integrated assessment (SYRINA) of endocrine disrupting chemicals. (DOCX 124 kb)

## Footnotes

1 DistillerSR® (https://distillercer.com/) is a proprietary project management tool for tracking studies through the screening process and storing data extracted from these studies using user-customized forms. Rayyan is a free web-based application (http://rayyan.qcri.org/) for screening and storing information culled from studies. Health Assessment Workspace Collaborative (HAWC): A Modular Web-based Interface to Facilitate Development of Human Health Assessments of Chemicals. https://hawcproject.org/.
