# Supplementary material for: A proposed framework for the systematic review and integrated assessment (SYRINA) of endocrine disrupting chemicals
Source: Environ Health. 2016 Jul 14;15:74. doi: 10.1186/s12940-016-0156-6 (PMC4944316; doi:10.1186/s12940-016-0156-6)

**Additional file 1: A proposed framework for the systematic review and integrated assessment (SYRINA) of endocrine disrupting chemicals**

**Table S1**. List of additional literature that may be useful to search strategy for systematic review

| Chemical / Toxicology | California EPA [Toxicity Criteria Database](http://www.oehha.ca.gov/tcdb/index.asp)  CHE [Toxicant and Disease](http://www.healthandenvironment.org/tddb) Database  EPA [ACToR](http://actor.epa.gov/actor/faces/ACToRHome.jsp) (Aggregated Computational Toxicology Resource)  EPA [Chemical Data Access Tool](http://java.epa.gov/oppt_chemical_search/)  EPA [Health & Environmental Research Online](http://hero.epa.gov/) (HERO)  EPA [Integrated Risk Information System](http://www.epa.gov/iris/search_keyword.htm) (IRIS)  EPA [Toxicity Reference Database](http://actor.epa.gov/toxrefdb/faces/Home.jsp) (ToxRefDB)  [ExPub](http://www.ebscohost.com/expub) (includes RTECS) – subscription  [National Toxicology Program](http://ntpserver.niehs.nih.gov/main_pages/NTP_ALL_STDY_PG.html) Study Status and Results  [PAN Pesticide](http://www.pesticideinfo.org/) Database  [PubChem](https://pubchem.ncbi.nlm.nih.gov/)  [Toxnet](http://toxnet.nlm.nih.gov/) (includes CCRIS, DART, Genetox, HSDB, IRIS, ITER)  [SciFinder](https://scifinder.cas.org/) – subscription  [TSCATS](http://yosemite.epa.gov/oppts/epatscat8.nsf/ReportSearch?OpenForm) |
| --- | --- |
| Clinical | [CenterWatch Clinical Trials](http://www.centerwatch.com/clinical-trials/listings/)  [ClinicalTrials.gov](https://clinicaltrials.gov/)  [Cochrane Central Register of Controlled Trials](http://www.cochrane.org/editorial-and-publishing-policy-resource/cochrane-central-register-controlled-trials-central) –subscription  [Current Controlled Trials](http://www.controlled-trials.com/)  [EU Clinical Trials Register](https://www.clinicaltrialsregister.eu/)  [WHO International Clinical Trials Registry](http://apps.who.int/trialsearch/) |
| Grey Literature | [DART-Europe](http://www.dart-europe.eu/basic-search.php) (E-Theses)  [Grey Literature Report](http://www.greylit.org/home)  [OAIster](http://oaister.worldcat.org/)  [Open Access Theses and Dissertations](http://oatd.org/)  [OpenDOAR](http://www.opendoar.org/)  [Registry of Open Access Repositories](http://roar.eprints.org/)  [Virtual Health Library](http://regional.bvsalud.org/php/index.php?lang=en) |
| Occupational Health | International Labour Organization [CISDOC](http://www.ilo.org/dyn/cisdoc2/cismain.home)  [National Institute for Occupational Safety and Health](http://www.cdc.gov/niosh/) (NIOSH)  [NIOSHTIC2](http://www2a.cdc.gov/nioshtic-2/default.asp)  [European Agency for Safety and Health at Work](https://osha.europa.eu/en)  [Labor Occupational Health Program Library](http://opac.libraryworld.com/opac/home)  [Occupational Safety and Health Administration](https://www.osha.gov/) (OSHA) |
| Regional Biomedical Databases | [African Index Medicus](http://indexmedicus.afro.who.int/)  [Latin American and Caribbean Health Science Information](http://lilacs.bvsalud.org/en/) (LILACS)  [Western Pacific Rim Index Medicus](http://wprim.org/) (WPRIM) |
| Systematic Reviews | [Cochrane Library](http://www.thecochranelibrary.com/view/0/index.html)  [Database of Promoting Health Effectiveness Reviews](http://eppi.ioe.ac.uk/webdatabases/Intro.aspx?ID=2) (DoPHER)  [Prospero](http://www.crd.york.ac.uk/PROSPERO/) |

| Table S2: Overview of methods for evaluation of individual human epidemiology and wildlife studies with possible use for EDCs: | | | | |
| --- | --- | --- | --- | --- |
| **Evaluation method and reference** | **General description** | **Details of evaluation criteria** | **Outcome of the evaluation** | **Possibility for EDC specific considerations** |
| The International Agency for Research on Cancer (IARC) approach for evaluation of carcinogenic risk to humans  IARC, 2006 | - Presents scientific principles and procedures used in developing IARC Monographs - Available at <http://monographs.iarc.fr/ENG/Preamble/index.php> | See Preamble Section A2 for detailed guidance.  Study quality should be assessed by considering the possible roles of bias, confounding and chance.  Causality should be evaluated using the Bradford Hill viewpoints. | No study-by-study quantitative score. | Can be used for EDCs; consider modifications as needed. |
| Navigation Guide  Developed at University of California, San Francisco  Woodruff and Sutton, 2011 | - Addresses risk of bias in specified domains | 7 domains   - recruitment, blinding, confounding, exposure assessment, incomplete outcome data, selective reporting, other bias, conflict of interest | - 4-level rating for each domain | Although not developed for EDCs specifically, the tool can be customized with modification of domains and additional questions relating to EDC-related issues.  Consideration of time window of exposure and timing of measurement of outcome assessed for a set of studies is included under “indirectness”. |
| Office of Health Assessment and Translation (OHAT)  Developed at U.S. National Institute of Environmental Health Sciences (NIEHS)  NTP (National Toxicology Program), 2015 | - Uses parallel approach to evaluate risk of bias from human, animal, and *in vitro* studies across evidence streams. - Has individual questions designated as applicable to specific types of human studies (e.g., controlled trial, case-control, cohort) - Updating guidance for assessing observational studies to be consistent with ACROBAT-NRSI, but tailored for exposure studies - Current version available at: <http://ntp.niehs.nih.gov/pubhealth/hat/noms/index-2.html> | 11 questions covering 7 domains (1-2 question per domain)   - selection, confounding, performance (in controlled exposure studies), attrition/exclusion, detection bias related to exposure characterization and outcome assessment, selective reporting, and other threats to bias   6 domains and 7 questions apply to observational human studies (e.g., cohort, case-control, cross-sectional studies) | - 4-level rating for each domain - Includes tiering approach for overall study evaluation and allows consideration of direction of bias | Although not developed for EDCs specifically, the tool can be customized with modification of domains and additional questions relating to EDC-related issues.  Consideration of time window of exposure and timing of measurement of outcome assessed for a set of studies is included under “indirectness.” |
| ACROBAT-NRSI  Developed by Cochrane Collaboration.  Sterne et al., 2014 | - ACROBAT-NRSI developed for non-randomised studies of clinical interventions; other methods adapted from tools used in clinical trials - Addresses risk of bias in specified domains - Current version available at: <https://sites.google.com/site/riskofbiastool/home> | 7 domains   - confounding, participant selection, measurement of intervention, departure from intended intervention, missing data, outcome measurement, selective reporting - Signaling and follow-up questions, developed for specific types of studies (cohort and case-control) | - 4-level rating for each domain - Include rating for overall study evaluation and incorporates direction of bias into domain (and overall) rating | Although not developed for EDCs specifically, the tool can be customized with modification of domains and additional questions relating to EDC-related issues. |
| Office of Report on Carcinogens (ORoC)  Developed at U.S. National Institute of Environmental Health Sciences (NIEHS)  NTP (National Toxicology Program), 2015 | - Method develop for epidemiology - Considers direction and impact of bias/limitation if possible - Current version available at: <https://ntp.niehs.nih.gov/pubhealth/roc/handbook/index.html> | - 6 risk of bias domains examining   - selection and attrition bias  - exposure misclassification (information bias); includes time window of exposure  - outcome misclassification (information bias); includes timing or measurement of outcome  - potential for confounding  - analysis  - selective reporting   - Additional domain for study sensitivity   Series of signaling and following questions used to provide a rating for a core question for each domain | - Rating of overall evaluation of the ability of each study to inform the hazard evaluation | Can be used for EDCs; consider modifications as needed. |

# Table S3: Example evaluation domains for human epidemiology and wildlife studies of EDCs. These domains are not specific to EDCs, but must be considered in assessment of EDC studies.

| Domains | Issues in Epidemiology and Wildlife Studies |  | |
| --- | --- | --- | --- |
| Population | - Is there evidence that participation (or follow-up) is jointly dependent on exposure and disease? - Are there measures of population abundance and diversity (e.g. ways to avoid selection bias)? | | |
| Exposure measure | - How well does the exposure measurement reflect the relevant time window(s)? | |  |
| Outcome measure | - Was the outcome ascertained with a relevant method, applied in the same manner in all participants, without knowledge of exposure status? - Is the measure a sensitive and valid measure of the outcome of interest? | |  |
| Confounding | - Does the analysis (or design) account for any strong risk factor that is also associated with the outcome in the study population? | | |
| Data analysis | - Does the analysis appropriately address missing data, modeling assumptions, and exposure-response patterns? | | |
| Selective Reporting | - Is a comprehensive set of results presented? | | |
| Sensitivity | - Is there any other aspect of the design, not otherwise discussed in the preceding questions, that limits the “sensitivity” of the study (i.e., the ability to detect an association)? | | |

| **Table S4: Overview of methods for evaluation of individual *in vivo* studies with possible use for EDCs:** | | | | |
| --- | --- | --- | --- | --- |
| **Evaluation method and reference** | **General description** | **Details of evaluation criteria** | **Outcome of the evaluation** | **Possibility for EDC specific considerations** |
| Navigation Guide  Developed at University of San Francisco  Woodruff and Sutton, 2011 | Navigation Guide developed for *in vivo* toxicity studies by adapting tool used by Cochrane/GRADE; criteria based on areas empirically tested within arena of human clinical trial research. | Navigation Guide: 7 domains (7 questions):  - sequence generation, allocation concealment, blinding, incomplete outcome data, selective reporting, conflict of interest, other | 4-level rating for each question: definitely low, probably low, probably high, definitely high risk of bias | Although not developed for EDCs specifically, the tool can be customized with additional questions relating to relevance or other EDC-related issues. Some aspects of relevance considered at different points in the evaluation process.  Consideration of time window of exposure and timing of measurement of outcome assessed for a set of studies under “indirectness”. |
| Science in Risk Assessment and Policy (SciRAP)  Developed at Stockholm University and Karolinska Institutet, Sweden.  Beronius et al. 2014b | - Developed for *in vivo* toxicity studies, including those not conducted according to standardized test guidelines - Criteria based on OECD test guidelines, and on previously published evaluation methods. Developed to fit the European regulatory system. - Web-based color-coding tool developed to aid evaluation. - Criteria and the color-coding tool are available free of charge at [www.scirap.org](http://www.scirap.org) | - Each study is evaluated for reliability and relevance - Evaluation of reliability divided into two tiers; all criteria in Tier I have to be fulfilled before continuing to more detailed Tier II evaluation - The criteria cover different areas of reliability: reporting, purpose, test compound, animals housing and feed, administration of test substance, measurement/data collection, statistics, discussion - Each reliability criterion rated according to 5-level rating system: not determined, not applicable, not fulfilled, partially fulfilled, fulfilled - 8 questions on relevance intended as guidance when evaluating relevance of study | 4 evaluation categories for reliability and relevance, respectively:   1. reliable/relevant without restrictions 2. reliable/relevant with restriction 3. not reliable/relevant 4. not assignable   (Method for categorization is under development) | Although not developed for EDCs specifically this method includes EDC-related criteria, e.g:   - Cage and bedding materials free of hormonally active substances - Contaminants such as phytoestrogens minimized in feed and water - Appropriate dose levels - A sensitive animal model is used - Sensitive and appropriate endpoints - Timing of exposure and measurement of endpoints are appropriate for study objectives |
| SYRCLE  Hooijmans et al. 2014 | Based on Cochrane Risk of Bias tool, adapted for animal intervention studies | 6 domains (10 questions):  - Selection (including comparison of baseline characteristics of animals), performance (including randomization of housing), detection, attrition, reporting, other | 3-level rating for each question: high, low, or unclear risk of bias | Although not developed for EDCs specifically, the tool can be customized with additional questions relating to relevance or other EDC-related issues. Some aspects of relevance considered at different points in the evaluation process.  Consideration of time window of exposure and timing of measurement of outcome assessed for a set of studies under “indirectness” |
| Criteria for Reporting and Evaluating ecotoxicity Data (CRED)  Developed at Stockholm University, RIVM and Swiss Centre for Applied Ecotoxicology  Moermond et al., 2015 | - Developed for aquatic ecotoxicity studies, including those not conducted according to standardized test guidelines - Criteria based on experiences from risk assessors, previous methods and OECD guidelines, developed to fit the European regulatory system. - When compared to the Klimisch method in a ring test with 75 risk assessors, the CRED evaluation method was the preferred method, and resulted in more transparent and consistent evaluations. - Can be used with the color-coding tool available at [www.scirap.org](http://www.scirap.org). | - Each study is evaluated for its reliability and relevance - 20 reliability criteria covering different areas: test setup, compound, organism, exposure conditions, statistical design and biological response. - 13 relevance criteria covering two areas: biological relevance, exposure relevance.   Each reliability criterion is rated according to a 4-level rating system: fulfilled, not fulfilled, not applicable, not reported | 4 evaluation categories for reliability and relevance, respectively:  1) reliable/relevant without restrictions,  2) reliable/relevant with restriction,  3) not reliable/relevant,  4) not assignable | Although not developed for EDCs specifically this method includes criteria that consider:   - Sensitivity and sex of the test species - Relevance of the endpoints e.g. mode of action - Timing of exposure and adequate duration - Spacing between exposure concentrations - Confounding factors such as stable conditions during the performance of experiments |
| Office of Health Assessment and Translation (OHAT)  Developed at U.S. National Institute of Environmental Health Sciences (NIEHS)  NTP (National Toxicology Program), 2015 | OHAT tool uses parallel approach to evaluate risk of bias from human, animal, and *in vitro* studies to facilitate consideration of risk of bias across evidence streams with common terms and categories.   - Current version of tool available at <http://ntp.niehs.nih.gov/pubhealth/hat/noms/index-2.html> | 6 domains (9 questions)s:  - selection, performance, attrition/ exclusion, detection bias related to exposure characterization and outcome assessment, selective reporting, and any other treats | 4-level rating for each question: definitely low, probably low, probably high, definitely high risk of bias; 3-level “tiering” approach based on specific criteria can be used to characterize overall study quality | Although not developed for EDCs specifically, the tool can be customized with additional questions relating to relevance or other EDC-related issues. Some aspects of relevance considered at different points in the evaluation process:  Consideration of time window of exposure and timing of measurement of outcome assessed for a set of studies under “indirectness” |
| Office of Report on Carcinogens (ORoC)  Developed at U.S. National Institute of Environmental Health Sciences (NIEHS)  NTP (National Toxicology Program), 2015 | - Method develop for animal toxicology - Considers direction and impact of bias/limitation if possible   Current version available at: <https://ntp.niehs.nih.gov/pubhealth/roc/handbook/index.html> | - 6 domains:   - study design, exposure conditions, outcome assessment and measurement, potential for confounding, analysis and selective reporting   - Each domain consists of risk of bias and sensitivity signaling and follow-up questions | - Rating of overall evaluation of the ability of each study to inform the hazard evaluation | Can be used for EDCs; consider modifications as needed |

# Table S5: Overview of methods for evaluation of individual *in vitro* studies with possible use for EDCs:

| **Evaluation method and reference** | **General description** | **Details of evaluation criteria** | **Outcome of the evaluation** | **Possibility for EDC specific considerations** |
| --- | --- | --- | --- | --- |
| Office of Health Assessment and Translation (OHAT)  Developed at U.S. National Institute of Environmental Health Sciences (NIEHS) | Uses parallel approach to evaluating risk of bias from human, animal, and *in vitro* or mechanistic studies to facilitate consideration of risk of bias across evidence streams with common terms and categories. Current version of tool available at <http://ntp.niehs.nih.gov/pubhealth/hat/noms/index-2.html> | \| 6 domains (9 questions) apply to in vitro studies: selection, performance, attrition/exclusion, detection bias related to exposure characterization and outcome assessment, selective reporting, and any other threats \| \| --- \| | - 4-level response option system:4 definitely low, probably low, probably high, definitely high risk of bias - 3-level “tiering” approach based on specific criteria can be used to characterize overall study quality | Although not developed for EDCs specifically, some aspects of relevance considered at different points in the evaluation process:  Consideration of time window of exposure and timing of measurement of outcome assessed for a set of studies under “indirectness”  The tool can be customized with additional questions relating to relevance or other EDC-related issues |

**Figure S1:**


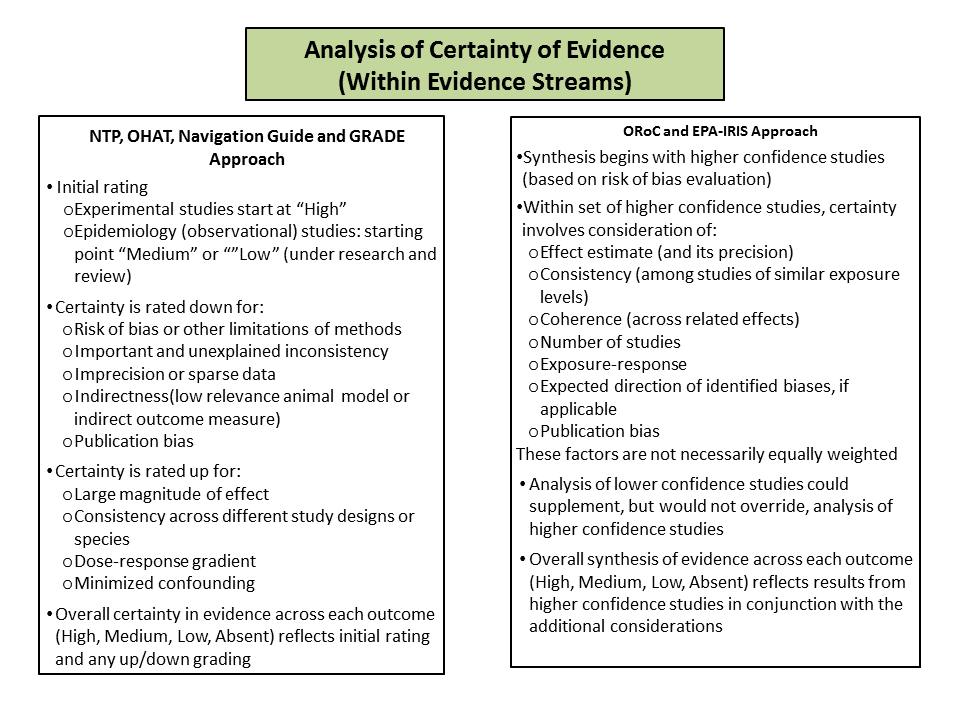

Supplement: Additional file 1: — A proposed framework for the systematic review and integrated assessment (SYRINA) of endocrine disrupting chemicals. (DOCX 124 kb) [file 12940_2016_156_MOESM1_ESM.docx]
